# Coronary artery disease, its associations with ocular, genetic and blood lipid parameters

**DOI:** 10.1038/s41433-023-02703-9

**Published:** 2023-08-16

**Authors:** Indrė Matulevičiūtė, Vacis Tatarūnas, Vilius Skipskis, Ieva Čiapienė, Audronė Veikutienė, Vaiva Lesauskaitė, Olivija Dobilienė, Dalia Žaliūnienė

**Affiliations:** 1 Department of Ophthalmology, Lithuanian University of Health Sciences, Kaunas, Lithuania; 2 Institute of Cardiology, Lithuanian University of Health Sciences, Kaunas, Lithuania; 3 Department of Cardiology, Lithuanian University of Health Sciences, Kaunas, Lithuania

**Keywords:** Metabolic disorders, Predictive markers

## Abstract

**Background/objectives:**

To investigate the associations between ophthalmic parameters, *CYP4F2* (rs2108622) and *ABCA1* (rs1883025) polymorphisms and coronary artery disease, considering the accessibility, non-invasive origin of retinal examination and its possible resemblance to coronary arteries.

**Subjects/methods:**

Overall 165 participants divided into groups based on the coronary angiography results and clinical status: control group (*N* = 73), MI group (*N* = 63), 3VD (three vessel disease) (*N* = 24). All the participants underwent total ophthalmic examination – optical coherence tomography (OCT) and OCT angiography of the macula region were performed and evaluated. Total cholesterol, high-density lipoprotein, low-density lipoprotein and triglyceride cholesterol (Tg-C) were tested. A standard manufacturer’s protocol for *CYP4F2* (rs2108622) and *ABCA1* (rs1883025) was used for genotyping with TaqMan probes.

**Results:**

GCL+ layer was thicker in control group vs. 3VD group (74.00; 62.67–94.67 (median; min.-max.) vs. 71.06; 51.33–78.44, *p* = *0.037*). T allele carriers under *ABCA1* rs1883025 dominant model were shown to have ticker retina and smaller foveal avascular zone in superficial capillary plexus and smaller Tg-C concentration. *ABCA1* rs1883025 was associated with retinal thickness (OR = 0.575, 95% CI 0.348–0.948, *p* = *0.030*). Univariate logistic regression showed that *ABCA1* rs1883025 CT genotype is associated with decreased risk for coronary artery disease development under overdominant genetic model (OR = 0.498, 95% CI 0.254–0.976; *p* = *0.042*) and codominant genetic model (OR = 0.468, 95% CI 0.232–0.945, *p* = *0.034*).

**Conclusions:**

Results of this study confirmed that non-invasive methods such as OCT of eye might be used for identification of patients at risk of CAD.

## Introduction

Coronary artery disease (CAD) is one of the main causes of mortality and disability in adult population. Studies investigating possible associations between eyes and heart diseases have been going on for decades, only the methods evolved with upcoming technologies [1–6]. At present the researchers suggest retinal investigation as a possible method to predict CAD [7, 8].

Cardiovascular diseases, such as CAD, stroke and heart failure, has been shown to correlate with the structure of retinal vessels, but the results are diverse and further studies are needed to develop a risk-scoring system using retinal vessels [9]. Atherosclerosis is a progressive chronic inflammatory condition involved in pathogenesis of CAD, stroke, and peripheral arterial disease [10]. Clinical studies show a direct link between immune cells, hyperlipidemia, atherosclerosis, and cardiovascular events as well as retinal diseases [11–13]. Thus, according to a novel description of atherosclerosis mechanism, the atherogenic process starts with the accumulation of several plasma lipoproteins in the subendothelial space at sites of flow perturbation and endothelial dysfunction [12, 13].

Lipids are not only a significant factor for atherosclerosis development but also an important component in retinal pigment epithelium (RPE) and photoreceptors function of the retina. The balance between production and anabolism of lipids is a key factor in retinal health. Several proteins on RPE surface are participating the influx (LDL receptor, VLDL receptor, SCARB1) and eflux (ABCA1, ABCG1) of lipids. Degraded lipids from photoreceptors outer segments are influxed in RPE and reused. Some of lipids, such as arachidonic acid (AA) are used to produce signalling lipids that are used to ensure proper function of the retina [14].

It was shown by several studies that 20-hydroxytetraenoic acid (20-HETE) has a significant role in cardiovascular disease. 20-HETE is a metabolite of AA. In human body AA is ω-hydroxylated *via* CYP450 pathway into 20-HETE [15]. 20-HETE has complex interactions with renin-angiotensin-aldosterone system (RAS). Both 20-HETE and the RAS work towards vascular dysfunction, hypertension, and inflammation [16]. 20-HETE was also shown to play a role in endothelium activation and dysfunction [17]. Studies with 20-HETE inhibitors in vascular inflammation/injury models showed that inhibition in 20-HETE reduces inflammatory reaction to injury [18, 19].

The *CYP4F2* SNP rs2108622 (namely, V433 M, G1347A, −1347 C > T and G20597A) was proved to lead to a protein with significantly reduced AA metabolising activity [20, 21]. The latter SNP has been investigated in the hypertensive patients [22, 23], stroke [24], myocardial infarction [23].

ATP-binding cassette transporter A1 (ABCA1) is an important protein that maintains cholesterol homoeostasis and is also significant in inflammatory response. ABCA1 interacts with lipid-free or poorly lipidated apolipoproteins (apos) to form nascent high-density lipoprotein (HDL) particles. ABCA1 can also interact with small HDL particles to accept sterols from cells [25]. ABCA1 in macrophages is extremely important in foam cell formation [26, 27]. It serves as an atheroprotector in macrophages not only through cholesterol efflux but also through its ability to modulate the inflammatory response [25]. It is thought that accumulation of lipids in subretinal space and increased inflammatory response are key factors in drusen formation and AMD development [28].

The proatherogenic cytokines (interferon (IFN)-γ, interleukin (IL)-1β, platelet-derived growth factor (PDGF)) have been shown to inhibit the expression of ABCA1 while anti-inflammatory cytokines such as IL-10 and TGF-β1 have been shown to increase the expression of ABCA1 [29]. The expression of ABCA1 is also upregulated when cell is overload with cholesterol [30].

The *ABCA1* SNP rs1883025 was shown to have impact on HDL cholesterol (HDL-C) concentration and A allele was associated to decreased HDL-C concentration [31, 32]. It was found that higher number of C alleles was associated with higher serum HDL-C level [33]. A large (3066 Caucasian subjects) cohort study has shown that T allele was protective against large and intermediate drusen and advanced AMD [34], however, it was not proven by a metanalysis [35]. Animal study including ABCA1/ABCG1 knock out mice showed increased lipid accumulation in RPE cells leading to RPE atrophy and inflammatory cells accumulation [36].

A recent review suggests that retinal photographs could be used in deep learning techniques for prediction of systemic diseases, including cardiovascular disease, while retinal optical coherence tomography (OCT) and optical coherence tomography angiography (OCT-A) are being used in prediction of cardiovascular disease in non-deep learning studies [37]. Considering the accessibility, non-invasive origin of retinal examination and its possible resemblance to coronary arteries we aimed to investigate the associations between ophthalmic parameters, *CYP4F2* (rs2108622) and *ABCA1* (rs1883025) polymorphisms and CAD.

## Methods

**Fig. 1:**
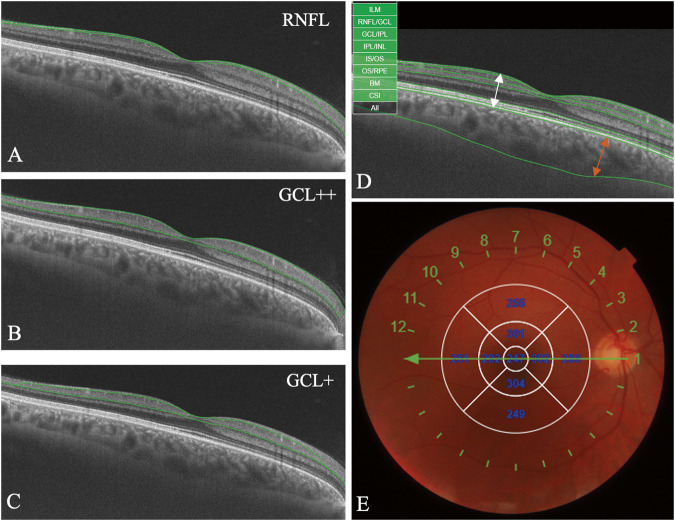
Optical coherence tomography of the macular region. **A**–**C** images represent the RNFL, GCL + + and GCL+ layers respectively. The layers are marked in between the green lines. The white and orange arrows in image **D** represents total retinal and choroidal thickness, respectively. **E** Device generated an Early Treatment of Diabetic Retinopathy Study (ETDRS) thickness map centred on the fovea. The ETDRS plot includes three circles with diameters of 1, 3 and 6 mm, dividing the macula into two rings and further dividing it into four quadrants: superior, inferior, nasal, and temporal.

One hundred and sixty-five participants from the Cardiology Department of the Hospital of the Lithuanian University of Health Sciences Kaunas Clinics were included in the study during the period between January 2019 and November 2021. All participants underwent ECG registration, echocardiography, and coronary angiography procedure. Based on the coronary angiography results and clinical status of the patient were formed groups: i) control group with coronary arteries without obstructions, ii) MI group, iii) 3VD (three vessel disease) group with all three coronary arteries affected and without previous history of acute coronary syndrome or revascularization. The patient selection and inclusion process are presented in supplements Fig. 1. The study was approved by the Kaunas regional bioethics committee (BE-2-101, 2018-12-20). Written informed consent was obtained from participants to allow analysis of the collected data. The study was planned in accordance with the Tenets of the Declaration of Helsinki.

**Systemic exclusion criteria** were the same for all groups. Patients with previous coronary angiography and percutaneous coronary intervention, diabetes, oncologic diseases, previous heart surgery, stroke were not included in the study. **Ophthalmic exclusion criteria** for all groups were high refractive error (myopia and hyperopia greater than 6.0 dioptres or astigmatism greater than +/− 3.0 dioptres), amblyopia, previous ocular trauma, intraocular inflammation, ocular surgeries except cataract removal with uneventful phacoemulsification surgery, glaucoma, macular disorders, or any conditions obscuring the view of the fundus.

The participants were asked to complete the questionnaire related to their cardiological condition. The questions included systolic and diastolic blood pressure measurement at home, previous cardiologic procedures – heart surgery, percutaneous coronary interventions, antihypertensive, cholesterol-lowering medications. According to the patients answers and patient case information, the participant was stated to have hypertension if the diagnosis was present in the case, or the patient was using antihypertensive medications. The waist circumference was measured [38]. Total cholesterol, HDL-C, LDL cholesterol (LDL-C) and triglyceride cholesterol (Tg-C) were tested as a standard treatment procedure for all the patients. The blood sample was drawn in an outpatient setting for control and 3VD group and after the hospitalisation for MI patients.

All the patients underwent total ophthalmic examination (visual acuity assessment, tonometry, biomicroscopy, ophthalmoscopy) in the Department of Ophthalmology of the Hospital of the Lithuanian University of Health Sciences Kaunas Clinics. The examination was performed during the first five days after myocardial infarction for MI group, within several weeks after coronary angiography for control and 3VD groups. The results of the optical coherence tomography (OCT) of the macula region were evaluated. The results are presented in nine Early Treatment of Diabetic Retinopathy Study (ETDRS) [39] regions (central, inner, and outer superior, inferior, nasal and temporal) by the OCT device. The average of the nine regions was calculated and presented as an average total thickness. The average total retinal, choroidal thickness, thickness of retinal layers (RNFL ((retinal nerve fibre layer), GCL + + (between internal limiting membrane (ILM) and inner plexiform layer-inner nuclear layer (INL) boundaries), GCL+ (between retinal nerve fibre layer-ganglion cell and inner plexiform layer-inner nuclear layer boundaries) were calculated. The images of macular OCT evaluation are presented in Fig. 1. The vascular density (VascDen) (6 × 6 mm) of the superficial (SCP) and deep vascular layers (DCP) as well as foveal avascular zone area (3 × 3 mm) was obtained from macular optical coherence tomography angiography. Full description of patient inclusion and examination process was described in previous publication [40].

Blood samples for genotyping were collected during first 5 days after MI, and several days after coronary angiography in the patients of 3VD and control groups. Genotyping procedures were performed at the certified Laboratory of Molecular Cardiology, Institute of Cardiology, Lithuanian University of Health Sciences, Kaunas, Lithuania. DNA extraction was performed by using the salting-out method. A standard manufacturer’s protocol for *CYP4F2* (rs2108622) and *ABCA1* (rs1883025) was used for genotyping with TaqMan probes (Applied Biosystems (ABI, Waltham, Massachusetts, USA). Genotyping was performed with Quantstudio 3 (ThermoFisherScientific) real-time PCR instrument.

### Statistical analysis

Statistical analysis was performed using IBM SPSS Statistics 27. To predict CAD with margin error of 5% and confidence level of 95% according to the incidence of CAD worldwide (1.72%) [41] the sample size of 26 patients in the group was calculated. The normal distribution of data was not observed, therefore nonparametric Kruskal–Wallis, and Mann–Whitney *U* tests were used for comparison of three (MI, healthy, 3VD groups) and two groups (healthy and CAD (MI and 3VD together), respectively. Multi-comparison Bonferroni adjusted *p* value was displayed when pairwise comparisons within three groups were performed. The *χ*^*2*^ test was used to compare the distribution of drug usage between groups. Binary logistic regression was used to predict CAD. The result was presented as an odds ratio (OR) with a 95% confidence interval (95% CI). Hardy-Weinberg equilibrium (HWE) was used to evaluate the observed and expected frequencies of variants of interest (rs2108622, rs1883025) between groups (MI, healthy, 3VD) using the *χ*^*2*^ test. The *χ*^*2*^ test was used to compare the distributions of *CYP4F2* (rs2108622) and *ABCA1* (rs1883025) between groups. Various genetic models (codominant (wild type homozygous versus heterozygous versus minor allele homozygous), recessive (wild type homozygous + heterozygous versus minor allele homozygous), dominant (wild type homozygous versus heterozygous + minor allele homozygous), overdominant (wild type homozygous + minor allele homozygous versus heterozygous), and additive inheritance) were used in the analysis. Quantitative variables were described as median; minimum-maximum, qualitative variables were described using frequencies and percent. A *p*-value < 0.05 was considered statistically significant.

## Results

A total of 73 healthy subjects, 68 patients with MI (53 STEMI and 15 non STEMI) and 24 patients with 3VD were included in the analysis. Table 1 represents demographic and clinical characteristics of study participants. Most of patients with 3VD had arterial hypertension (*p* = *0.034*). Systolic blood pressure values were similar in all groups, while diastolic blood pressure was highest in MI group (pairwise comparison Control vs. MI *p* = *0.013, Bonferroni corrected p* = *0.040*, MI vs. 3VD *p* = *0.042, Bonferroni corrected p* = *0.125*). The difference of body mass index after pairwise comparison was determined between Control and MI groups only (*p* = *0.004, Bonferroni corrected p* = *0.012)*. Total cholesterol and Tg-C blood levels were similar. The difference between HDL-C and LDL-C was determined between MI and 3VD groups after pairwise comparison (*p* = *0.018, Bonferroni corrected p* = *0.053; p* = *0.007, Bonferroni corrected p* = *0.022*, respectively). The usage of cholesterol lowering drugs differed significantly between groups (31.5% vs. 7.5% vs. 54.2% in control, MI and 3VD groups respectively, *p* < *0.001*). Echocardiography parameters did not differ significantly between the groups except ejection fraction, where MI group showed significantly lower ejection fraction after pairwise comparison (MI vs. control p < 0.001, MI vs. 3VD p < 0.001, *Bonferroni corrected p* < *0.001* in both comparisons) (Table 1).

**Table 1:** Demographic and clinical characteristics of study participants.

Characteristic	Control group	MI group	3VD group	*p value*
Sex, n (%)				
Male	40 (54.8)	41 (60.3)	17 (70.8)	*0.374*
Female	33 (45.2)	27 (39.7)	7 (29.2)	
Age, years (median; min.-max.)	59.22; 42.52–75.68	58.89; 35.51–77.21	64.87; 49.98–74.57	*0.407*
Arterial hypertension, n (%)	62 (84.9)	50 (73.5)	23 (95.8)	***0.034***
Systolic blood pressure, mmHg (median; min.-max.)	136.5; 100–175	138; 80–250	135; 120–160	*0.609*
Diastolic blood pressure, mmHg (median; min.-max.)	80; 60–102	85; 60–140	80; 70–100	***0.023***
Body mass index, kg/m^2^ (median; min.-max.)	29.70; 23.03–45.91	28.06; 20.55–38.37	29.05; 22.07–36.0	***0.016***
Waist circumference, cm (median; min.-max.)	101; 81–132	99; 70–127	104; 76–113	*0.276*
Total cholesterol, mmol/l (median; min.-max.)	5.46; 3.33–8.53	5.77; 3.12–9.90	5.86; 3.40–14.10	*0.241*
HDL-C, mmol/l (median; min.-max.)	1.36; 0.60–2.65	1.21; 0.61–2.06	1.44; 0.88–4.23	***0.028***
LDL-C, mmol/l (median; min.-max.)	3.80; 0.87–5.84	3.85; 1.58–6.47	2.70; 0.9–6.47	***0.021***
Tg-C, mmol/l (median; min.-max.)	1.26; 0.44–3.82	1.39; 0.46–11.69	1.36; 0.73–12.1	*0.517*
Left ventricular end diastolic diameter, mm (median; min.-max.)	49.5; 41.0–73.0	48.0; 37.0–61.0	48.0; 37.0–56.0	*0.143*
Left ventricular mass index, g/mm^2^ (median; min.-max.)	98.51; 70,12–182.70	95.46; 47.91–176.06	86.82; 65.05–141.13	*0.324*
Ejection fraction, % (median; min.-max.)	55; 25–70	45; 20–55	55; 50–60	**<0.001**

Bold values represent statistically significant differences.

### Ophthalmic evaluation

The visual acuity and intraocular pressure of the patients were similar in all the groups and within normal limits. Three patients had cataract surgery performed and monofocal intraocular lens implanted in their study eye, while all the other participants had light to moderate cataract. The results of OCT and OCTA investigation are displayed in Table 2. None of the ophthalmic parameters differed significantly between groups, except GCL+ thickness. After a pairwise comparison a statistically significant difference of GCL+ thickness was observed between control and 3VD groups (*p* = *0.012, Bonferroni corrected p* = *0.037*). GCL+ is a complex of ganglion cell layer and inner plexiform layer (IPL) where the bodies of first order neurons of visual pathway are located and connective cells of IPL. This complex is extremely important in visual function and is mostly investigated in glaucomatous optic neuropathy [42].

**Table 2:** Ophthalmic examination results of the control, MI and 3VD groups.

Characteristic	Control group	MI group	3VD group	*p* *value*
	Median; min.-max.	Median; min.-max.	Median; min.-max.	
Retinal thickness, µm	285.56; 258.67–323.67	281.11; 204.0–313.67	281.67; 250.67–297.11	*0.078*
Choroidal thickness, µm	258.67; 132.33–390.44	251.11; 67.44–414.22	208.00; 32.11–405.89	*0.114*
RNFL thickness, µm	30.44; 25.44–46.78	30.94; 24.78–64.78	30.72; 26.22–102.44	*0.479*
GCL + + thickness, µm	105.33; 90.22–134.11	105.17; 79.11–135.89	102.78; 85.22–115.67	*0.331*
GCL+ thickness, µm	74.00; 62.67–94.67	71.33; 60.89–89.22	71.06; 51.33–78.44	***0.024***
FAZ area SCP, µm^2^	283.71; 0–611.28	299.36; 48.85–886.29	312.76; 93.87–580.43	*0.795*
FAZ area DCP, µm^2^	231.50; 41.57–716.22	255.94; 44.12–700.84	362.72; 86.31–760.61	*0.112*
Central vascular density SCP	20.58; 10.87–27.72	19.99; 7.60–27.38	18.70; 11.75–26.47	*0.462*
Central vascular density DCP	18.68; 9.31–27.15	18.68; 6.82–28.19	18.83; 10.70–26.84	*0.745*

*FAZ* foveal avascular zone, *SCP* superficial capillary plexus, *DCP* deep capillary plexus.

Bold values represent statistically significant differences.

### CYP4F2 rs21086226 and ABCA1 rs1883025 variants in represented patient groups

The distribution of genotypes and alleles of *CYP4F2* rs21086226 and *ABCA1* rs1883025 in the control, MI and 3VD group patients is displayed in supplements Table 1. There was no statistically significant difference between distributions under different genetic models. Hardy-Weinberg equation-based quality assessment of the genotypes showed no deviation in any of the analysed groups in both SNPs (*p* > 0.05).

### CYP4F2 rs2108622 and ABCA1 rs1883025 variants, ophthalmic parameters, and lipid profile

Genetic models of inheritance were used to determine the effect of *CYP4F2* and *ABCA1* variants on clinical parameters. Differences between central VascDen medians (min.-max.) in DCP and *CYP4F2* rs21086226 in C allele carriers and non-carriers were observed (18.92 (6.82–29.05) vs. 17.18 (14.50–18.51), *p* = *0.036*). DCP is a vascular plexus, providing highly oxygenated blood to plexiform layers, that consists of high number of synapses and is essential for proper retinal function. In a study of *Genevois O* et al. DCP was proved to form collateral drainage in venous occlusive diseases [43].

Significant differences were observed when total retinal, retinal layers thickness and Tg-C concentration were compared under *ABCA1* rs1883025 dominant model (Table 3). No differences related to VascDen measurements were observed. No differences were observed under other genetic models (supplements Tables 2, 3), except SCP FAZ area under overdominant model.

**Table 3:** Ophthalmic and blood test results in T allele carriers of ABCA1 rs1883025.

Parameter	Non T allele carriers	T allele carriers	*p* *value*
	Median; min.-max.	Median; min.-max.	
Retinal thickness, µm	282.0 (256.11–323.67)	282.55 (204.00–315.78)	***0.042***
GCL + + thickness, µm	103.89 (84.00–134.11)	105.11 (79.11–119.67)	***0.034***
GCL+ thickness, µm	71.22 (59.89–94.67)	72.55 (60.89–86.67)	***0.015***
FAZ area SCP, µm^2^	312.803 (92.549–886.289)	260.244 (0–611.279)	***0.008***
Tg-C, mmol/l	1.43 (0.46–12.1)	1.23 (0.44–3.41)	***0.034***

*FAZ* foveal avascular zone, *SCP* superficial capillary plexus, *DCP* deep capillary plexus.

Bold values represent statistically significant differences.

### Regression model for coronary artery disease

Univariate logistic regression showed that *ABCA1* rs1883025 CT genotype is associated with decreased risk for CAD development under overdominant genetic model (OR = 0.498, 95% CI 0.254–0.976; *p* = *0.042*) and codominant genetic model (OR = 0.468, 95% CI 0.232–0.945, *p* = *0.034*). Total retinal thickness and GCL+ was also related to slightly decreased risk of CAD development (Table 4).

**Table 4:** Univariate logistic regression of ABCA1 rs1883025 and CYP4F2 rs21086226 for coronary artery disease.

	OR	CI 95%	*p* *value*
*CYP4F2* rs21086226			
**Codominant model**			
CC			
CT	0.911	0.473–1.753	*0.780*
TT	1.974	0.366–10.651	*0.429*
**Dominant model**			
CC			
TT + CT	1.013	0.538–1.907	*0.967*
**Recessive model**			
TT			
CC + CT	2.040	0.384–10.833	*0.403*
**Overdominant model**			
CC + TT			
CT	0.875	0.458–1.672	*0.685*
**Additive model**	1.075	0.628–1.840	*0.791*
*ABCA1* rs1883025			
**Codominant model**			
CC			
CT	0.468	0.232–0.945	***0.034***
TT	1.221	0.380–3.929	*0.737*
**Dominant model**			
CC			
TT + CT	1.631	0.865–3.074	*0.130*
**Recessive model**			
TT			
CC + CT	1.530	0.489–4.784	*0.465*
**Overdominant model**			
CC + TT			
CT	0.498	0.254–0.976	***0.042***
**Additive model**	0.802	0.496–1.297	*0.368*
*Ophthalmic parameters*			
Retinal thickness	0.976	0.955–0.998	***0.035***
Choroidal thickness,	0.258	0.993–1.002	*0.258*
RNFL layer thickness	0.137	0.986–1.105	*0.137*
GCL + + layer thickness	0.972	0.939–1.006	*0.109*
GCL+ layer thickness	0.934	0.890–0.981	***0.006***
FAZ area SCP	1.001	0.999–1.003	*0.425*
FAZ area DCP	1.001	0.999–1.003	*0.509*

Bold values represent statistically significant differences.

## Discussion

In this study we tested hypothesis onassociations between ophthalmic parameters, lipid profile and CAD. We found that *ABCA1* rs1883025 is related to retinal thickness while *CYP4F2* rs2108622 is related to the VascDen in DCP. *ABCA1* rs1883025 CT genotype is associated with decreased risk for CAD development in all participants.

In the current study, we analysed average retinal, choroidal, and retinal layers thickness and found that GCL+ thickness was significantly higher in healthy group than 3VD group. In our previous study [40], we analysed ocular parameters in ETDRS segments and found significant segmental differences in total choroidal, retinal and GCL+ thicknesses. Other studies, investigating ocular parameters in CAD patients rarely performed retinal layer analysis. Wang et al. [44] analysed total retinal thickness and did not find any significant differences between healthy and CAD patients. They found a significant decrease in vessel density in CAD patients, opposite to our results. Neoh et al. [45] investigated RNFL thickness in 3VD patients and controls and found a significant decrease in RNFL thickness in 3VD group, while we only observed a slight decrease that was not statistically significant. Unfortunately, they did not analyse GCL + . *Xie H* et al. [46] evaluated hypertensive patients and found that ganglion cell layer was thinner in patients with higher blood pressure. GCL+ and specifically GCL consist of the first order visual neuron bodies that are important in visual signalling and glaucoma. One the mechanism related to normal tension glaucoma is microvascular dysfunction and oxidative stress [47] that are the key factors related to atherosclerosis and CAD. A study by Song et al. have also found a relation between glaucoma, mainly affecting RNFL and GCL and atherosclerosis [48]. When performing a genetic analysis, we found that the distribution of *CYP4F2* (rs2108622) and *ABCA1* (rs1883025) alleles in control group was similar to the results of control groups from other studies performed in Lithuania [49, 50]. The control groups consisted of patients without changes in the macular region of the retina. Genome-wide association study, representing 7 different population showed that the prevalence of rs1883025 A (T) allele in European-American population is 26% [51]. The Finnish study, investigating vit. E supplementation found that the prevalence of rs2108622 T (A) allele in male population is 19% [52]. Similarly, the prevalence of rs1883025 A (T) was 26.7% and rs2108622 T (A) was 20.5%.

CYP4F2 is a monooxygenase important in 20-HETE metabolism and inflammatory state of the body. Considering CAD, a chronic inflammatory condition we hypothesised to find different allelic distribution between groups. In our study we did not find any differences of *CYP4F2* (rs2108622) alleles distribution in healthy, MI and 3VD groups, similarly to *Al Eitan LN* et al. [53] where healthy and cardiovascular patients were compared. A study investigating Chinese Han population [54] found a significantly higher frequency of GG genotype in CAD patients than controls, but only a haplotype consisting of both rs2108622 and rs3093105 was found to be related to CAD development. A metanalysis performed by Zhang et al. [55] showed decreased risk of CAD development in *CYP4F2* (rs2108622) GA polymorphism group. Geng [56] did a meta-analysis investigating rs2108622 in hypertensive patients and found a strong link between hypertension and previously mentioned SNP. Our study included 81% of participants diagnosed with hypertension (no differences between groups, *p* > 0.05). This could be a factor impacting similar distribution of *CYP4F2* rs2108622 alleles between groups in our study.

The distribution of ophthalmic parameters in accordance with SNPs was analysed. We found that TT (rs2108622) carriers had significantly lower central VascDen in DCP. DCP is comprised of two vascular layers – intermediate at the IPL / inner nuclear layer (INL) boundary, and deep at the INL/outer plexiform layer boundary [57]. There are no studies evaluating retinal VascDen and *CYP4F2* rs2108622. A study performed by Mockute et al. [50] showed that the central retinal thickness was larger in CC carriers in comparison to CT/TT carriers, while we did not find any differences. CYP4F2 is involved in 20-HETE metabolism and chronic inflammation. Studies investigating ophthalmic inflammatory diseases [58, 59] and age-related macular degeneration that has an inflammatory pathogenetic pathway [60] showed a significant capillary rarefaction in the affected patients. We hypothesise that TT (AA) genotype is related to more profound inflammatory response and decreased VascDen because of that. Hormel et al. stated that retinal vascular pathologies include mostly DCP changes, which could be also related to atherosclerosis as a widespread vascular pathology [57].

We compared the distribution of *ABCA1* (rs1883025) alleles between healthy, MI and 3VD groups and found no significant differences, like Fouladseresht et al. [61] where two groups (healthy and CAD) were compared.

When retinal and blood test results under various models were analysed, we found significant differences under dominant model of *ABCA1* rs1883025. CC allele carriers were shown to have thinner total as well as GCL+ and GCL + + retinal thickness and significantly larger foveal avascular zone. All the aforementioned results are in accordance with each other. SCP provides blood supply to the ganglion cell complex that consists of RNFL, GCL and inner plexiform layer representing different structures of ganglion cells [57]. We could hypothesise that increase in FAZ represents decreased blood supply to the ganglion cell complex thus causing decrease in GCL+ and GCL + + as well as total retinal thickness. A study performed by Mockute et al. [50] investigated central retinal thickness in control group and AMD patients and did not find any differences between *ABCA1* rs1883025 various alleles carriers. In an animal model study ABCA1 was shown to be an important protein in lipid transport in retina. A mouse model lacking ABCA1 and ABCG1 showed lipid accumulation, retinal degeneration, and inflammation [36].

A study performed in Iranian population [61] found that the effect of *ABCA1* rs1883025 is only prominent in haplotype rs2422493/rs1800976/rs2230806/rs1883025 carriers, however we performed a binary logistic regression analysis and found that *ABCA1* rs1883025 CT genotype is associated with decreased risk for CAD development under overdominant genetic model. A Japanese study searching for genetic loci related to susceptibility to early onset CAD did not prove *ABCA1* rs1883025 to be related [62].

ABCA1 is a protein essential in intracellular cholesterol homoeostasis and reverse cholesterol transport [36, 62, 63]. We compared the concentrations of cholesterol according to rs1883025 allele distribution and found higher Tg-C concentration in CC carriers and interestingly did not find any differences in other lipids including HDL-C concentrations. Do [64] showed that previously mentioned SNP is significantly related Tg-C and LDL-C but did not show risk to CAD. *Teslovich TM* investigated European population and found relation between rs1883025 HDL-C and total cholesterol [65].Our study has limitations. The sample size for genetic analysis is rather small. The 3VD is significantly smaller than other groups included in the study. The initial enrolment of the participants was rather the same in all groups, however, 3VD group had significantly more comorbidities, such as stroke, diabetes, renal dysfunction, AMD, etc. resulting in their exclusion of the study.

## Conclusions

*ABCA1* rs1883025 was associated with retinal thickness. *CYP4F2* rs2108622 under recessive model (CC/CT vs. TT) may influence VascDen in DCP. *ABCA1* rs1883025 CT allele, retinal thickness and GCL+ may predict CAD. Results of this study confirmed that non-invasive methods such as OCT of eye might be used for identification of patients at risk of CAD. Large scale randomised studies are required to better understand the effect of biomarkers detected in this study.

Supplementary information is available at Eye’s website

## Summary

### What was known before:

- Cardiovascular diseases manifests with the structure of retinal vessels. However, the exact pathogenetic mechanism is not clearly described.
- ATP-binding cassette transporter A1 is crucial for reverse cholesterol transport and homoeostasis. It promotes the efflux of cholesterol from cells, thus it has anti-inflammatory and antiatherogenic activity.
- CYP4F2 is another important enzyme in metabolism of fatty acids. Alterations of CYP4F2 activity may have a significant role in control of inflammation.

### What this study adds:

- ABCA1 rs1883025 CT allele, retinal thickness and GCL+ may predict CAD.
- ABCA1 rs1883025 CC allele carriers have thinner total as well as GCL+ and GCL + + retinal thickness and significantly larger foveal avascular zone.

### Supplementary information

Supplements figure 1.

Supplements table 1.

Supplements table 2.

Supplements table 3.

## Data Availability

All data supporting the findings of this study are available within the paper and its Supplementary Information. Should any raw data files be needed they are available from the corresponding author upon reasonable request.
